# Epigallocatechin-3-gallate: a multi-target bioactive molecule derived from green tea against Oropouche virus—a computational approach to host–pathogen network modulation

**DOI:** 10.3389/fchem.2025.1590498

**Published:** 2025-07-03

**Authors:** Abdullah Al Noman, Pranab Dev Sharma, Umme Fathima Tuz Zohora, Farhana Akter Shifa, Emad M. Abdallah, Bader Y. Alhatlani

**Affiliations:** ^1^ School of Pharmacy, BRAC University, Dhaka, Bangladesh; ^2^ Biotechnology Program, Department of Mathematics and Natural Science, BRAC University, Dhaka, Bangladesh; ^3^ Department of Biology, College of Science, Qassim University, Qassim, Saudi Arabia; ^4^ Unit of Scientific Research, Applied College, Qassim University, Buraydah, Saudi Arabia

**Keywords:** Oropouche virus, epigallocatechin-3-gallate, molecular docking, antiviral agents, computational biology, lipophilicity GI absorption low

## Abstract

The Oropouche virus (OROV), an emerging arbovirus transmitted by arthropods, has caused significant outbreaks in South and Central America, with over half a million reported cases. Despite its public health threat, no approved vaccines or antiviral treatments exist for Oropouche fever (OF). This study explores the potential of epigallocatechin-3-gallate (EGCG), a bioactive polyphenol from green tea, as an antiviral agent against OROV using computational approaches. Due to the lack of experimentally resolved OROV protein structures, we employed AlphaFold2 to predict 3D models of key viral proteins, including RNA-dependent RNA polymerase (RdRp), envelopment polyprotein, nucleoprotein, and glycoprotein Gc. Molecular docking revealed strong binding affinities between EGCG and these targets, with particularly high interactions for RNA polymerase (−7.1 kcal/mol) and envelopment polyprotein (−8.7 kcal/mol), suggesting the inhibition of viral replication and entry. Protein–protein interaction (PPI) network analysis identified critical human host genes (e.g., FCGR3A, IRF7, and IFNAR1) involved in immune responses, while Gene Ontology (GO) and the Kyoto Encyclopedia of Genes and Genomes (KEGG) pathway analyses highlighted enriched antiviral and inflammatory pathways. ADMET profiling indicated challenges in EGCG’s bioavailability, including poor gastrointestinal absorption and blood–brain barrier permeability, but its low toxicity and natural origin support its potential as a lead compound. These findings suggest that EGCG may disrupt OROV infection through multi-target mechanisms, warranting further experimental validation. This study provides a foundation for developing EGCG-based therapeutics against OROV and underscores the utility of computational methods in antiviral drug discovery.

## 1 Introduction

Oropouche fever (OF) is caused by the Oropouche virus (OROV), which is transmitted through arthropod vectors (Sakkas et al., 2018). Recently, this lesser-known arbovirus has re-emerged on a significant scale, posing a global threat (Riccò et al., 2024). OROV belongs to the Peribunyaviridae family and was discovered first in Tobago and Trinidad (Benitez et al., 2024). Since then, it has led to outbreaks in several South and Central American countries, with more than half a million diagnosed cases (Zhang Y. et al., 2024). The actual number of cases is likely higher due to misdiagnosis as symptoms overlap with those of other febrile illnesses such as dengue, yellow fever, chikungunya, Zika, West Nile, and Guama (Riccò et al., 2024; Zhang Y. et al., 2024).

OROV persists in nature in two cycles—urban and sylvatic. In the urban cycle, the *Culicoides paraensis* midge is the main vector. Additionally, the *Culex quinquefasciatus* mosquito, which is prevalent in tropical regions, also transmits the virus by biting both humans and animals (Benitez et al., 2024). The OROV is distinguished by its negative-sense, single-stranded RNA genome, which is covered within a spherical lipid envelope (Zhang Y. et al., 2024). The genome consists of three single-stranded negative-sense RNA segments (large, medium, and small). The genome is composed of three segments of single-stranded negative-sense RNA: large, medium, and small. Sequencing analyses of the small segment have identified four distinct genotypes: I, II, III, and IV (Benitez et al., 2024). The helical nucleocapsid houses essential components such as the RNA-dependent RNA polymerase (RdRp), nucleocapsid protein (N), and viral surface glycoproteins (Gc) (Zhang Y. et al., 2024).

**FIGURE 1:**
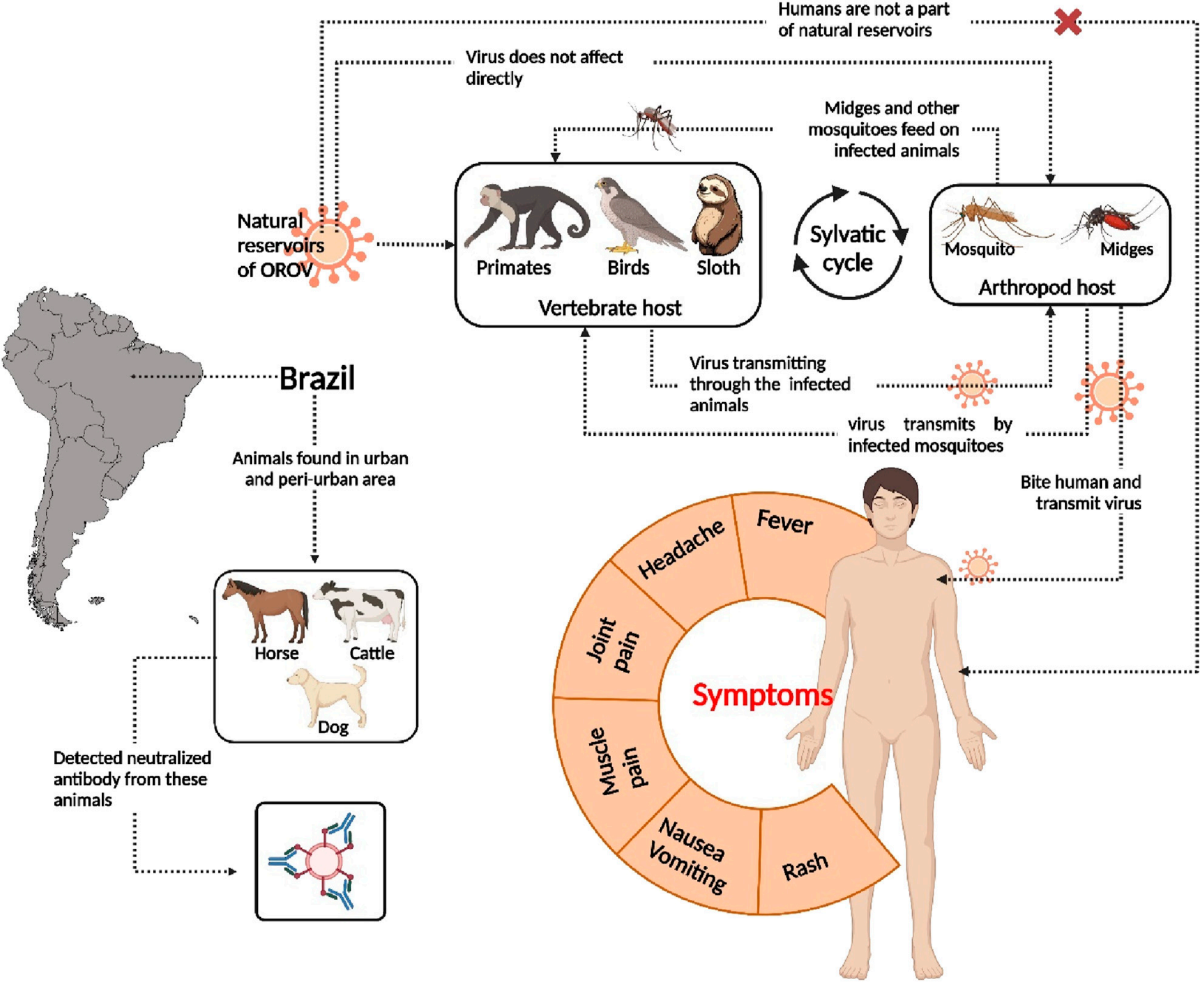
Sylvatic cycle of Oropouche virus, its symptoms, and detected animals for neutralized antibody.

Most cases of Oropouche fever are mild, with symptoms including headache, muscular pain, rash, and nausea (Figure 1). However, in some instances, the virus can cause more severe conditions such as encephalitis and meningitis (The Lancet Infectious Diseases, 2024). Despite its significant threat to public health, there are at present no approved vaccinations or specific antiviral treatments for Oropouche fever (Zhang Y. et al., 2024). This highlights the urgent need for effective therapeutic interventions.

Epigallocatechin-3-O-gallate (EGCG) is found in green tea (*Camellia sinensis*) as a predominant catechin (Kaihatsu et al., 2018). It is recognized as a powerful antioxidant that protects against oxidative damage in living organisms and also in food (Zhong et al., 2012). It has been studied for its antiviral properties against a wide range of viruses, including DNA viruses such as herpes simplex virus, hepatitis B virus, adenovirus, and human papillomavirus and RNA viruses such as dengue virus, chikungunya virus, Zika virus, hepatitis C virus (HCV), influenza virus, human immunodeficiency virus (HIV), and Ebola virus (Kaihatsu et al., 2018). Given its broad-spectrum antiviral capabilities, EGCG has been studied for Oropouche fever.

Oropouche fever has recently re-emerged on a large scale, posing a major public health concern. Again, over half a million diagnosed cases were reported in South and Central American countries. The actual number of cases may be underreported due to misdiagnosis as symptoms overlap with those of other febrile diseases. The point of concern is that approved vaccines are currently unavailable. Furthermore, there are no specific antiviral treatments for the fever, and comprehensive gene data and important protein structures of the Oropouche virus are currently unavailable. Therefore, homology modeling was used. This highlights the urgent need for effective antiviral treatments.

We identified EGCG (from green tea) as a potential antiviral agent against OROV as it has shown broad-spectrum antiviral capabilities against various DNA and RNA viruses. Thus, we performed molecular docking to assess its binding ability with viral proteins and identify important viral pathways to target. Furthermore, the binding affinities support EGCG’s potential as a drug candidate.

**FIGURE 2:**
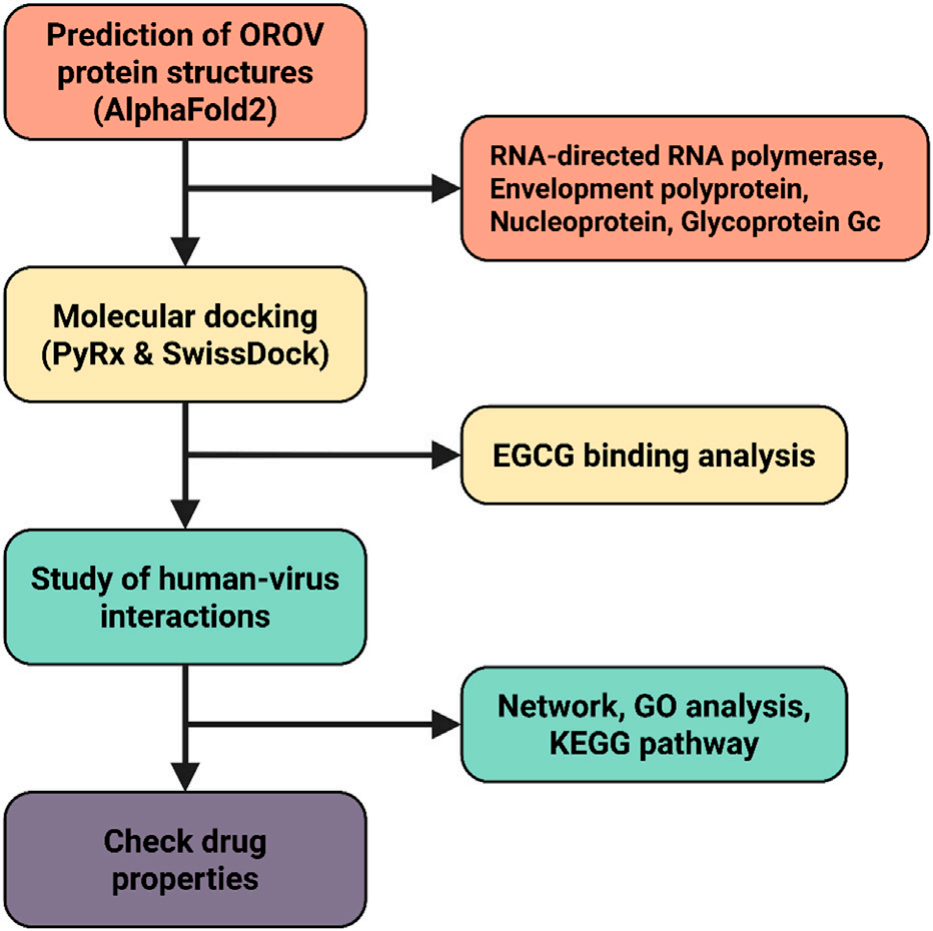
Detailed workflow of the investigation strategy for EGCG against the Oropouche virus.

In this study, we investigated EGCG, a bioactive polyphenol derived from green tea, as a potential antiviral candidate against OROV. EGCG has demonstrated broad-spectrum antiviral activity against diverse RNA and DNA viruses, including dengue, chikungunya, and Zika viruses, by interfering with viral entry, replication, and assembly. Using computational approaches, we evaluated the binding affinity of EGCG with essential OROV proteins, including RNA polymerase and glycoprotein Gc, to elucidate its mechanism of action. Our findings highlight EGCG’s promising interactions with viral targets and its potential to disrupt critical pathways in the OROV lifecycle. This study not only advances our understanding of EGCG’s antiviral properties but also provides a foundation for future experimental validation and therapeutic development against Oropouche virus infections. A schematic representation of the study’s workflow is provided in Figure 2.

## 2 Materials and methods

### 2.1 Protein modeling

Due to the lack of available 3D structures for these proteins, we predicted their structural conformations (Forrest et al., 2006). The protein sequences were collected from two primary sources: UniProt and NCBI. Three protein sequences—RNA-dependent RNA polymerase, envelopment polyprotein, and nucleoprotein—were retrieved from UniProt (https://www.uniprot.org/, accessed on 16 May 2025), ensuring standardized annotation and high-quality reference data (Apweiler et al., 2009). The fourth sequence, glycoprotein Gc, was obtained from NCBI (https://www.ncbi.nlm.nih.gov/, accessed on 16 May 2025) (Sayers et al., 2019). All sequences were acquired in the FASTA format, maintaining consistency for further processing and analysis. The OROV protein structures were predicted using the AlphaFold2 Collab platform (https://colab.research.google.com/github/sokrypton/ColabFold/blob/main/AlphaFold2.ipynb#scrollTo=AzIKiDiCaHAn, accessed on 16 May 2025), which is a cutting-edge deep learning tool for protein modeling. AlphaFold2 Collab employs a two-step process involving multiple neural network architectures (Yang et al., 2023). Initially, the system predicts residue distances and orientations, followed by a refinement step that incorporates additional structural details to enhance accuracy (Adiyaman et al., 2023). This platform enables efficient and precise protein structure prediction using advanced deep learning algorithms and extensive training datasets (Jumper et al., 2021). As a result, high-quality protein models were obtained, which are suitable for further analysis and interpretation.

### 2.2 Molecular docking

#### 2.2.1 Ligand selection and preparation

EGCG (PubChem CID: 65064) was used as a drug compound for its antiviral activity, and it is the significant polyphenolic catechin in green tea (Kaihatsu et al., 2018; Henss et al., 2021). This compound prevents the cell entry of different viruses, such as the influenza virus (Kim et al., 2013), chikungunya virus (Weber et al., 2015), human immunodeficiency virus (HIV) (Fassina et al., 2002; Yamaguchi et al., 2002; Williamson et al., 2006), and hepatitis C virus (HCV) (Ciesek et al., 2011; Calland et al., 2012; Chen et al., 2012; Colpitts and Schang, 2014). The structure of the compound was collected from PubChem (https://pubchem.ncbi.nlm.nih.gov/, accessed on 22 October 2024). First, the compound was imported into PyRx software. Next, the charge of that ligand was minimized, converted into the PDBQT format, and docked. After docking, the conformation with the lowest binding affinity was downloaded as a PDB file (Vázquez-Jiménez et al., 2022; Ayodele et al., 2023).

#### 2.2.2 Target selection and preparation

The PDB file was first loaded in ChimeraX 1.9 using the open filename.pdb command. The protein chains were then selected using select/A or refined using the ‘select protein’ command (Goddard et al., 2018). Non-protein components such as solvents, ligands, and ions were removed using the commands delete solvent, delete ligand, and delete ions. Next, the Dock Prep tool was used to add missing hydrogen atoms and assign atomic charges (assign charges), ensuring proper protonation states and electrostatic considerations for docking simulations (Pettersen et al., 2021). Once cleaned and prepared, the modified structure was saved using the save prepared_protein.pdb function. Finally, the processed protein was opened in Discovery Studio, and the structures were used to verify the presence of any remaining heteroatoms. If heteroatoms were detected, they were manually removed before saving the final version for computational analysis. This method ensured that the protein was well-prepared for molecular docking studies (Al Noman et al., 2024; Sharma et al., 2025).

#### 2.2.3 Identification of the active site and the grid box for protein–ligand interactions

The binding sites of each target protein were determined using Discovery Studio’s predictions, and the grid boxes were optimized based on the suggested binding regions (BIOVIA Discovery Studio, 2025). The identified active sites facilitated precise grid box adjustments tailored to the protein structure (Srinivasan et al., 2019; Wang et al., 2020; Naveed et al., 2024). Discovery Studio proved to be an effective tool for rapidly locating these sites (Alhawarri et al., 2023). To evaluate potential molecular interactions, the refined 3D structures of the target proteins and selected drug compounds were uploaded to PyRx for site-specific docking. The grid box was set to the appropriate dimensions, and the binding energy for each compound was calculated accordingly.

#### 2.2.4 Re-docking

To confirm docking results, SwissDock, a web-based docking tool using the EADock DSS algorithm, was used as an alternative approach (Grosdidier et al., 2011). This method helped verify the binding affinities of the selected compounds with target proteins, thus ensuring consistency with previous findings (Bugnon et al., 2024). For SwissDock, targets were directly uploaded in the PDB format, utilizing the tool’s built-in protein preparation feature (Bugnon et al., 2024). SMILES representations of compounds were inserted and processed within the platform. Grid box size and position were set based on active sites, which were identified earlier using Discovery Studio.

### 2.3 Virus–host connection and Gene Ontology analysis

#### 2.3.1 Identification of targets

The targets associated with the Oropouche virus were collected from the Online Mendelian Inheritance in Man (OMIM) database (https://www.omim.org/, accessed on 16 May 2025) and GeneCards database (https://www.genecards.org/, accessed on 16 May 2025) (Hamosh et al., 2005; Darif et al., 2021). The keywords “Oropouche virus” and “Oropouche fever” were set as search options in these databases. Related target symbols corresponding to the Oropouche virus were accumulated.

#### 2.3.2 PPI network construction

The STRING database (https://string-db.org/, accessed on 16 May 2025) was utilized to construct a protein–protein interaction (PPI) network among the identified intersecting targets, with *Homo sapiens* selected as the reference species (Szklarczyk et al., 2023). The generated interaction file was then processed and visualized using Cytoscape 3.10.3 software (Szklarczyk et al., 2023). Following this, the cytoHubba plugin in Cytoscape was employed to determine the top 20 highly connected targets within the network (Chin et al., 2014).

#### 2.3.3 Gene Ontology analysis

The DAVID online platform (https://davidbioinformatics.nih.gov/, accessed on 16 May 2025) and GeneCloudOmics (https://genecloudomics.bii.a-star.edu.sg/, accessed on 16 May 2025) was used to analyze Gene Ontology (GO) terms and Kyoto Encyclopedia of Genes and Genomes (KEGG) pathways for the intersection targets of the virus (Helmy et al., 2021; Sherman et al., 2022). The analysis included biological processes, cellular components, molecular functions, and KEGG pathways. Based on gene count values, the top 10 GO categories and 70 KEGG pathways were identified. For visualization, bioinformatics online tools (https://www.bioinformatics.com.cn/en, accessed on 16 May 2025) were applied (Tang et al., 2023). Additionally, overlapping genes underwent KEGG mapping analysis with a focus on *Homo sapiens*, covering pathways related to metabolism, environmental information processing, cellular processes, organismal systems, and human diseases.

### 2.4 ADMET analysis

ADMET analysis stands for absorption, distribution, metabolism, excretion, and toxicity assessment, which is crucial for drug development. It predicts how a drug behaves in the human body (Guan et al., 2018). Absorption identifies how the drug enters the bloodstream (Devadasu et al., 2018). Distribution determines where it travels within the body (Talevi and Bellera, 2023). Metabolism examines how the body breaks it down, and excretion focuses on how the drug and its by-products leave the body (Fernandez et al., 2011). Finally, toxicity predicts the potential harmful effects (Yao et al., 2009). A perfect ADMET profile ensures better efficacy and safety in new pharmaceuticals. To calculate the properties, an online tool named SwissADME (http://www.swissadme.ch/index.php, accessed on 16 May 2025) was used.

## 3 Results

### 3.1 Predicted protein models

**FIGURE 3:**
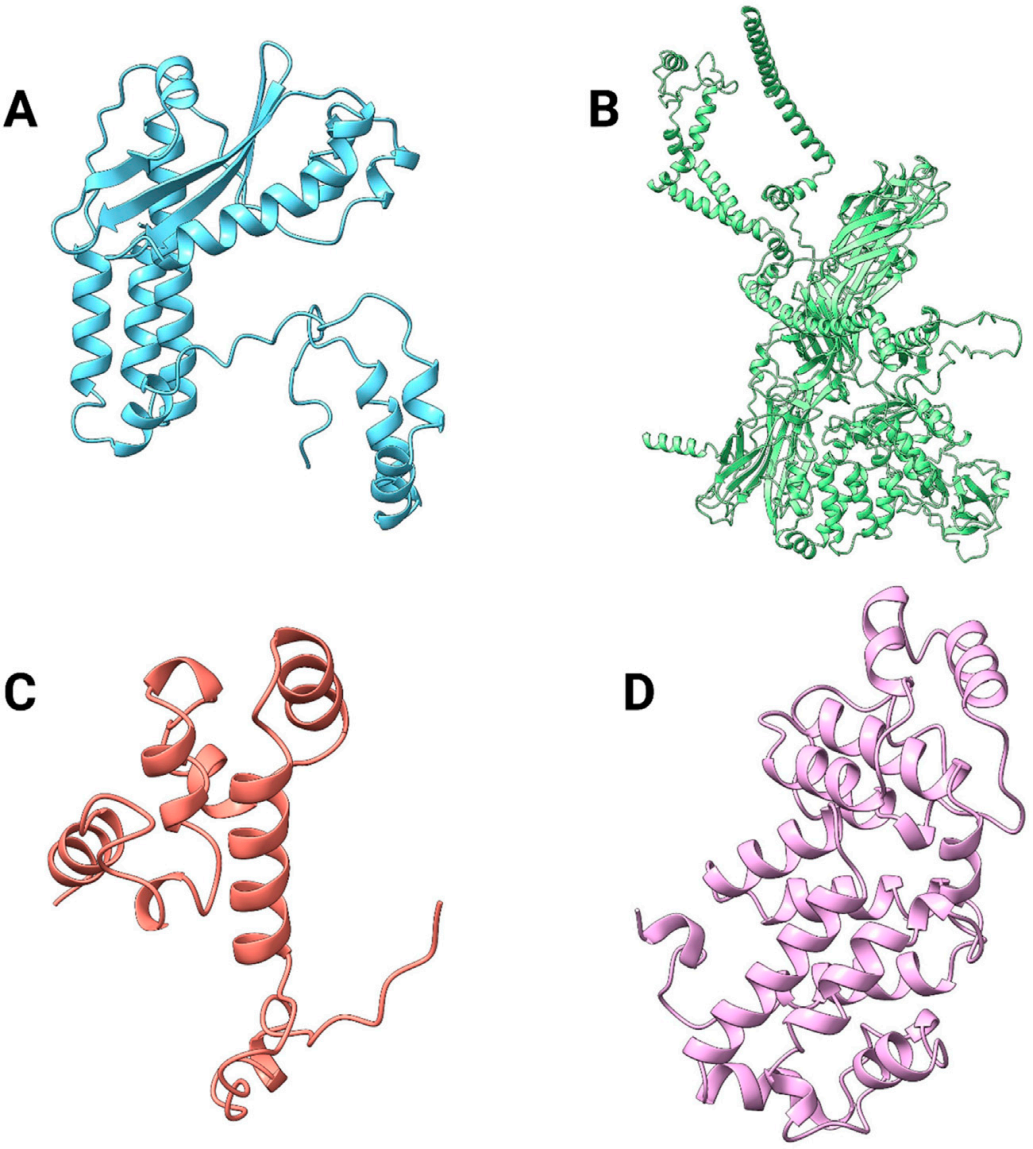
Visualization of four different proteins of the Oropouche virus predicted by AlphaFold2. **(A)** RNA-dependent RNA polymerase, **(B)** envelopment polyprotein, **(C)** nucleoprotein, and **(D)** glycoprotein Gc.

These viral proteins were selected due to their roles in viral genome replication, surface glycoprotein encoding and processing,and , virus assembly and budding and for functions in viral pathogenesis, replication, and transcription (Figure 3) (Orthobunyavirus, 2024; Travassos Da Rosa et al., 2017; Murillo et al., 2018; Gutierrez et al., 2020). These functions are essential for the virus to survive and infect a host, whether human or animal. For this reason, these proteins were targeted. The predicted protein structures obtained using the AlphaFold2 Collab platform yielded varying confidence scores based on plDDT and predicted template modeling (pTM) values. The RNA-dependent RNA polymerase exhibited a plDDT score of 88.1 and a pTM value of 0.715, indicating high structural reliability. The envelopment polyprotein demonstrated a plDDT score of 78.8 and a pTM value of 0.543, reflecting moderate confidence. Moreover, the nucleoprotein yielded a plDDT score of 93.2 and a pTM value of 0.808, supporting its structural accuracy. Finally, the glycoprotein Gc showed a plDDT score of 88.7 and a pTM value of 0.867, ensuring reasonable confidence in its predicted conformation. The plDDT score measures the confidence in the predicted local structure of each protein residue, ranging from 0 to 100, with higher values indicating greater reliability and structural accuracy (Carugo, 2023). Likewise, the pTM score assesses the degree of structural similarity between two folded protein models, ranging from 0 to 1. A pTM value above 0.5 suggests a significant resemblance, allowing for meaningful structural interpretations (Wuyun et al., 2024). Therefore, the obtained results confirm that the protein model is of high quality. The 3D structure of each protein is shown in Figure 3.

**FIGURE 4:**
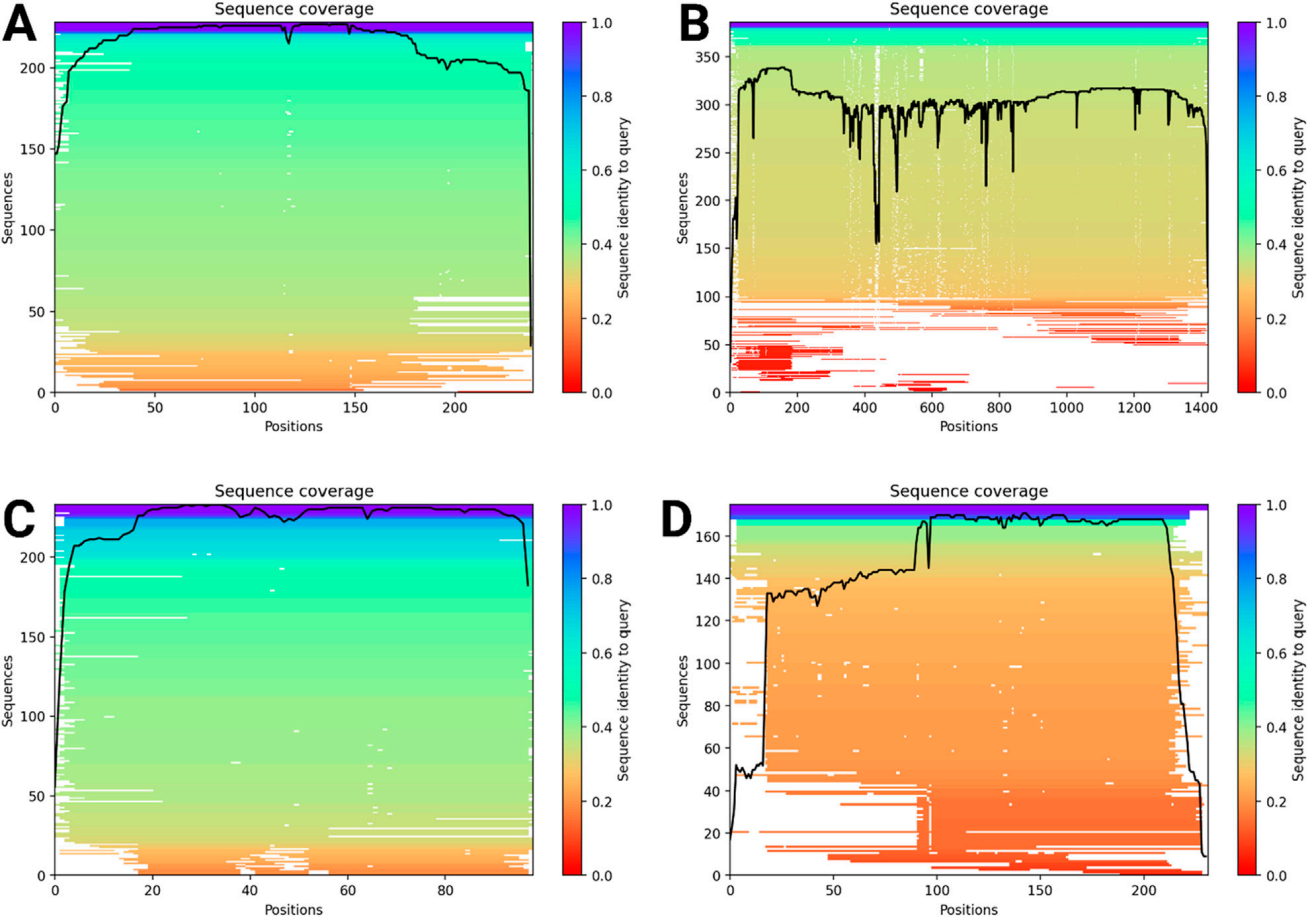
Sequence identity analysis across amino acid positions. Sequence coverage plots for four datasets **(A–D)** showing sequence positions on the x-axis and sequence counts on the y-axis. The color gradient represents the sequence identity, with red indicating low identity and blue indicating high identity. The black line illustrates coverage variations across different sequence positions. **(A)** RNA-dependent RNA polymerase, **(B)** envelopment polyprotein, **(C)** nucleoprotein, and **(D)** glycoprotein Gc.

Figure 4 consists of four graphs labeled A, B, C, and D, where each graph depicts a sequence identity across amino acid positions of the four different proteins. Figure 4A shows high sequence identity, with most positions above 0.5 and many exceeding 0.8. Figure 4B exhibits more fluctuation, with identity values varying yet generally remaining above 0.5. Figure 4C follows a similar pattern to Figure 4A, with consistently high identity values across most positions. Figure 4D starts with lower identity levels but progressively increases, stabilizing beyond 0.8 across the sequence. Occasional dips in all graphs indicate regions of variability in sequence conservation. The differences across graphs highlight conserved and variable regions within the protein. Overall, the sequence identity trends suggest moderate-to-high conservation in most positions. The presence of high identity values supports structural reliability and functional significance. These insights help assess sequence conservation, guiding further analysis.

**FIGURE 5:**
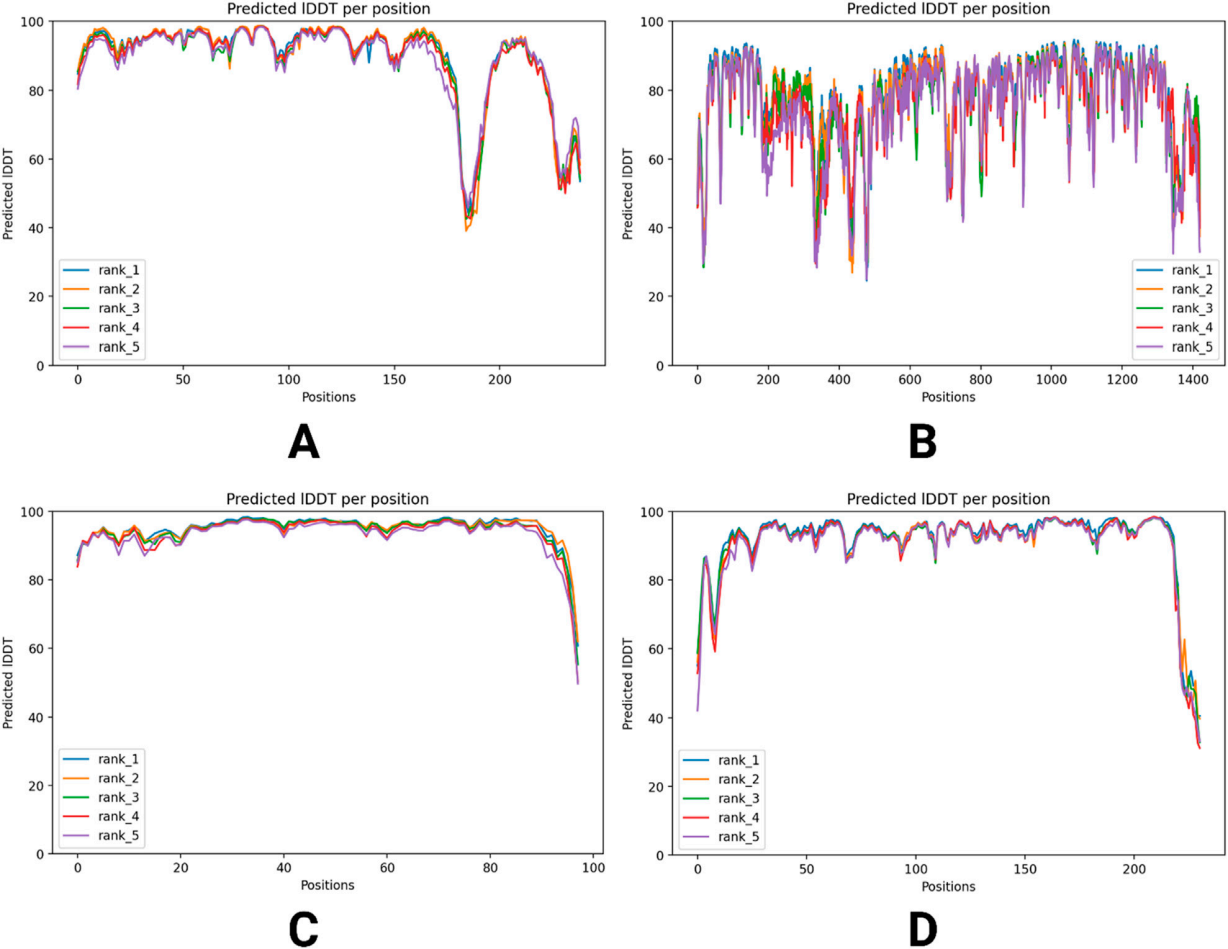
plDDT score distribution across four different predicted models of viral proteins. Predicted IDDT values for five ranks (rank_1 to rank_5) across four datasets **(A–D)**, illustrating sequence variability. The x-axis represents sequence positions, while the y-axis shows predicted IDDT values, indicating confidence in structural predictions. The graphs highlight variations in prediction reliability across different ranks and datasets. **(A)** RNA-dependent RNA polymerase, **(B)** envelopment polyprotein, **(C)** nucleoprotein, and **(D)** glycoprotein Gc.

Furthermore, Figure 5 presents the plDDT score distribution for each residue across the five predicted models, ranked from rank_1 to rank_5. The majority of residues exhibit plDDT scores above 80, indicating high confidence in their local structural accuracy. Notably, rank_1, the top-ranked model, displays the highest proportion of residues with elevated plDDT values, suggesting greater reliability in its prediction. The distribution of scores across the models highlights variations in structural confidence, with lower-ranked models showing minor fluctuations. Overall, the figure provides insight into the stability and precision of the predicted protein structures, reinforcing the quality of AlphaFold2’s computational modeling.

### 3.2 Ramachandran plot analysis

The Ramachandran plot is the graphical representation that indicates the phi (φ) and psi (ψ) angles of amino acids in a protein (Ho and Brasseur, 2005). The x-axis exhibits the phi angle, and the y-axis shows the psi angle (Priestle and Paris, 1996). The graph indicates distinctive regions where various phi and psi combinations are available. The important regions are the left-handed alpha helix, right-handed alpha helix, and beta-sheet.

**FIGURE 6:**
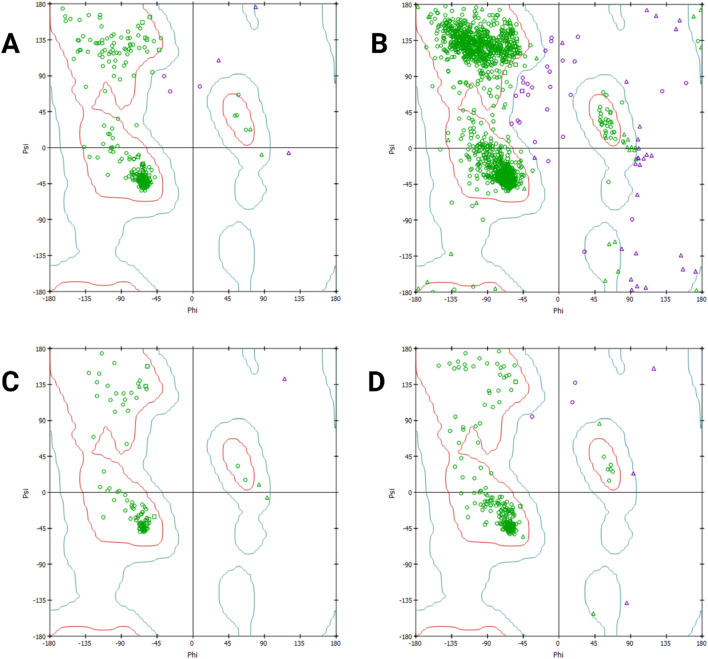
Ramachandran plot analysis for different predicted proteins. Ramachandran plot depicting the distribution of backbone dihedral angles (phi and psi) for protein structures. The x-axis represents phi angles, while the y-axis represents psi angles, with different regions highlighted as follows: allowed regions in blue, favored regions in green, and disallowed regions in red. The density of points indicates structural stability, with most residues clustering in energetically favorable conformations. **(A)** RNA-dependent RNA polymerase, **(B)** envelopment polyprotein, **(C)** nucleoprotein, and **(D)** glycoprotein Gc.

Figure 6A exhibits a high density of residues in favored regions, suggesting well-defined secondary structures, with minimal outliers in disallowed regions. Figure 6B shows a strong clustering within the allowed regions, though a few residues fall into disallowed areas, indicating structural flexibility. The protein in Figure 6C follows a similar trend, with clear concentrations in alpha-helical and beta-sheet conformations and showing fewer violations, thus reinforcing its structural stability. In Figure 6D, although the protein maintains its expected fold, deviations in loop regions suggest potential dynamic flexibility. Overall, the plots confirm that the proteins are structurally well-folded.

### 3.3 Docking result

#### 3.3.1 Active site prediction and grid box adjustment

**FIGURE 7:**
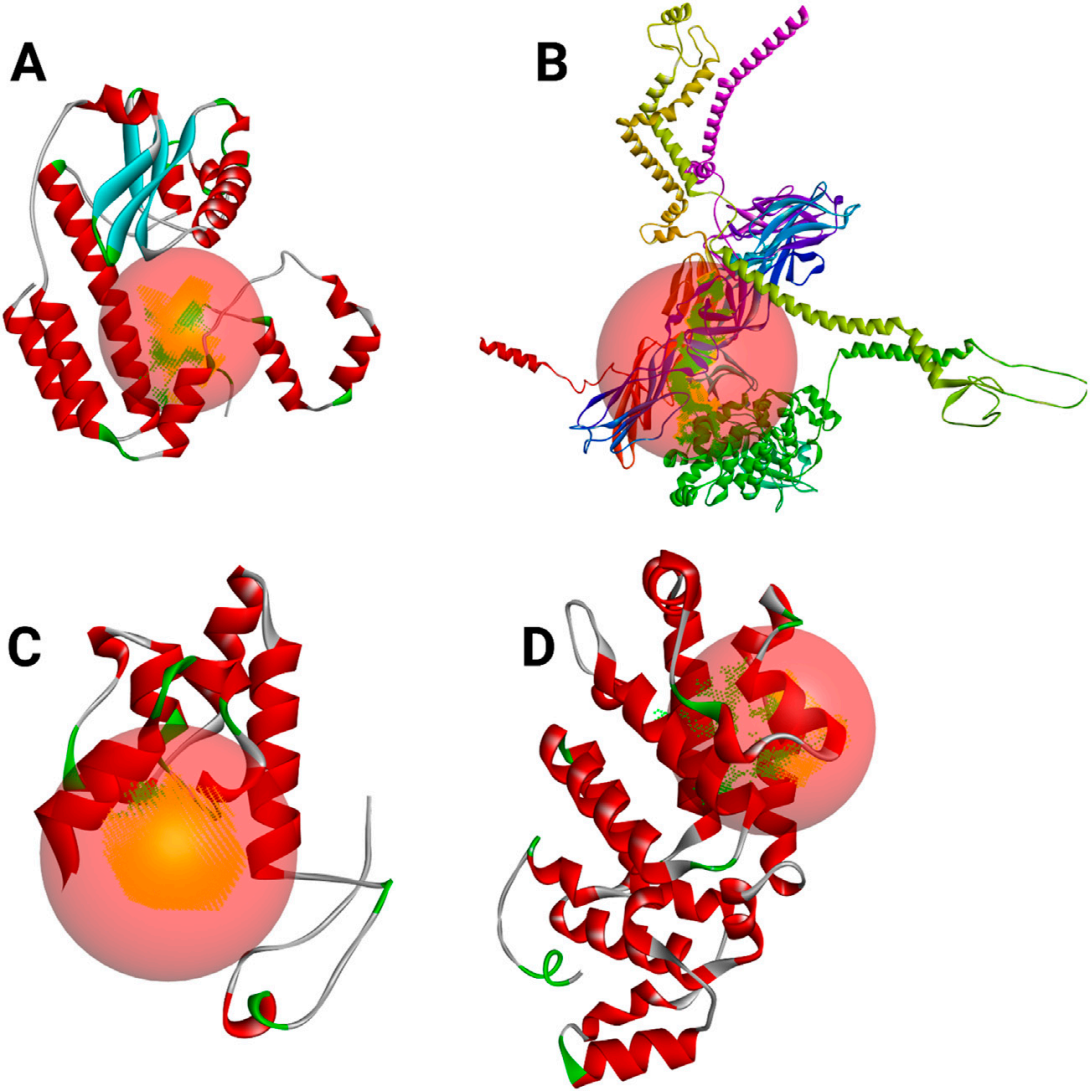
Predicted active sites for each identified target, with red circles highlighting the regions corresponding to these active sites. **(A)** RNA-dependent RNA polymerase, **(B)** envelopment polyprotein, **(C)** nucleoprotein, and **(D)** glycoprotein Gc.

Figure 7 displays the predicted active sites for RNA-dependent RNA polymerase, envelopment polyprotein, nucleoprotein, and glycoprotein Gc, which were identified through Discovery Studio. These binding regions highlight key residues involved in molecular interactions, aiding in structural and functional analysis.

For RNA-dependent RNA polymerase, the grid box was set into center-x = 12.2199, center-y = −14.6230, and center-z = −2.3110 and dimension-x = 14.8321, dimension-y = 16.2933, and dimension-z = 17.9138. The box was fit into center-x = 0.6775, center-y = 24.4438, and center-z = −7.6009 and dimension-x = 39.7578, dimension-y = 45.5983, and dimension-z = 37.6246 for envelopment polyprotein. The box was fit into center-x = −0.1471, center-y = −8.7348, and center-z = −2.8733 and dimension-x = 16.1054, dimension-y = 14.7307, and dimension-z = 18.2230 for nucleoprotein. For glycoprotein Gc, the box was measured into center-x = 4.5268, center-y = −1.1932, and center-z = −9.2487 and dimension-x = 22.8151, dimension-y = 20.6135, and dimension-z = 22.5106.

#### 3.3.2 Protein–ligand interaction

**FIGURE 8:**
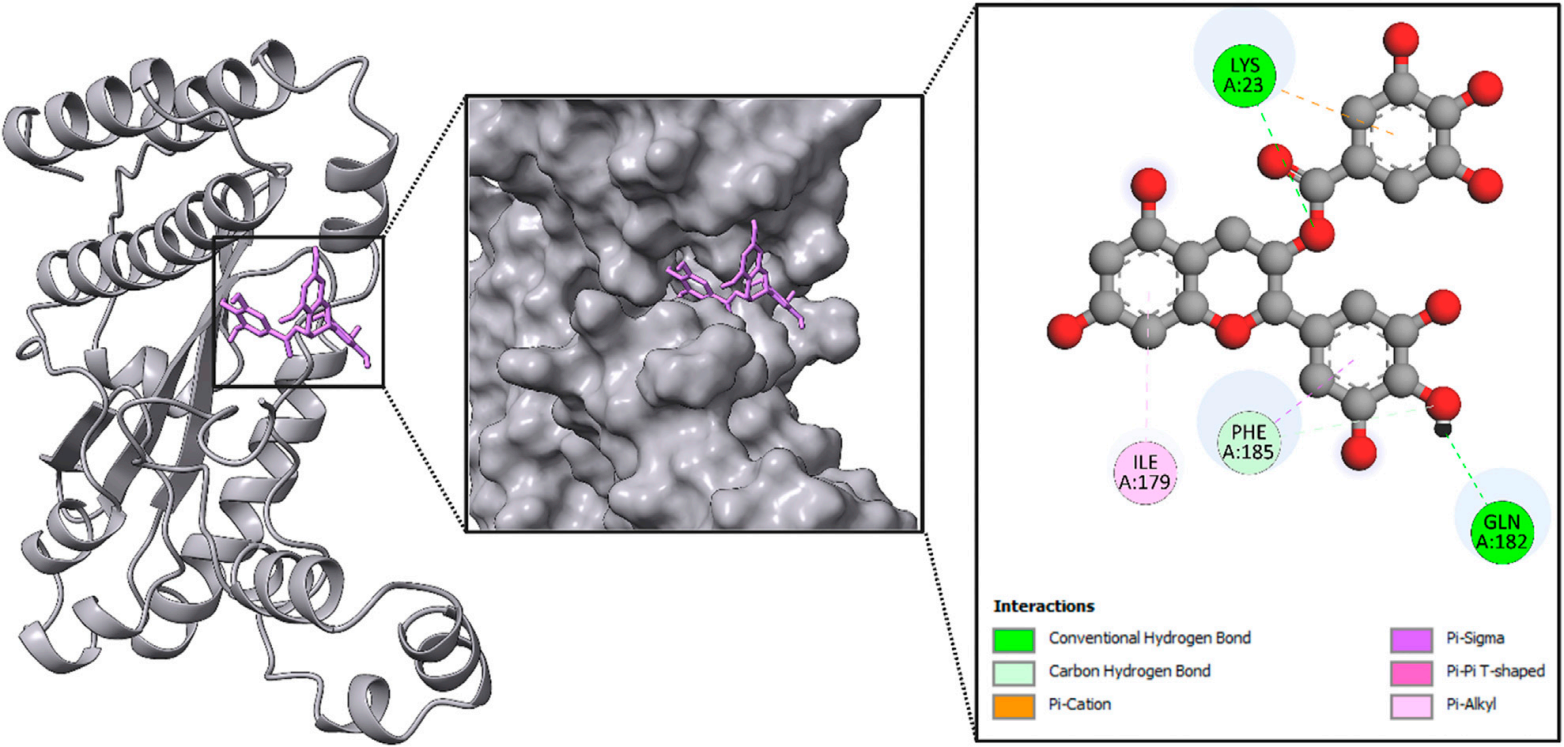
Structural analysis of the compound inside the protein (RNA-dependent RNA polymerase) and its interactions with proteins through different amino acids after docking.

**FIGURE 9:**
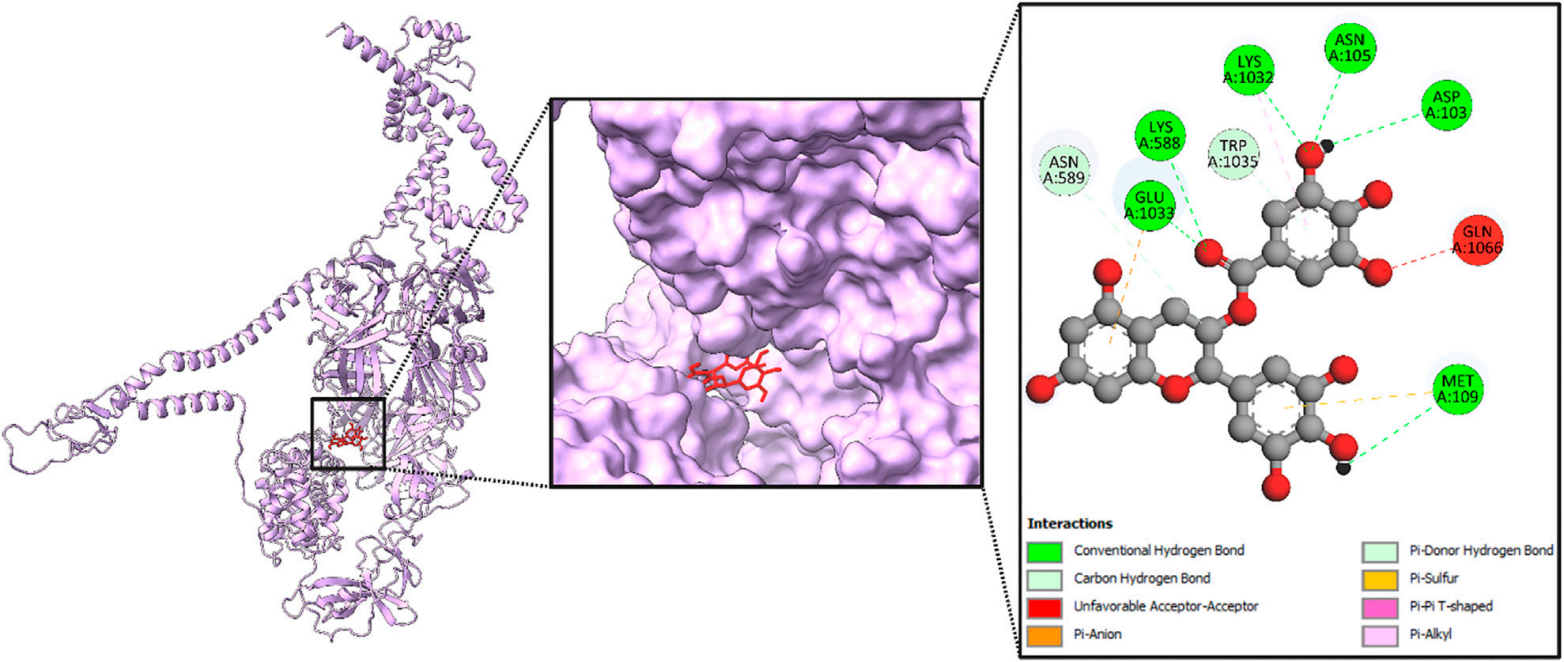
Structural analysis of the compound inside the protein (envelopment polyprotein) and its interactions with proteins through different amino acids after docking.

**FIGURE 10:**
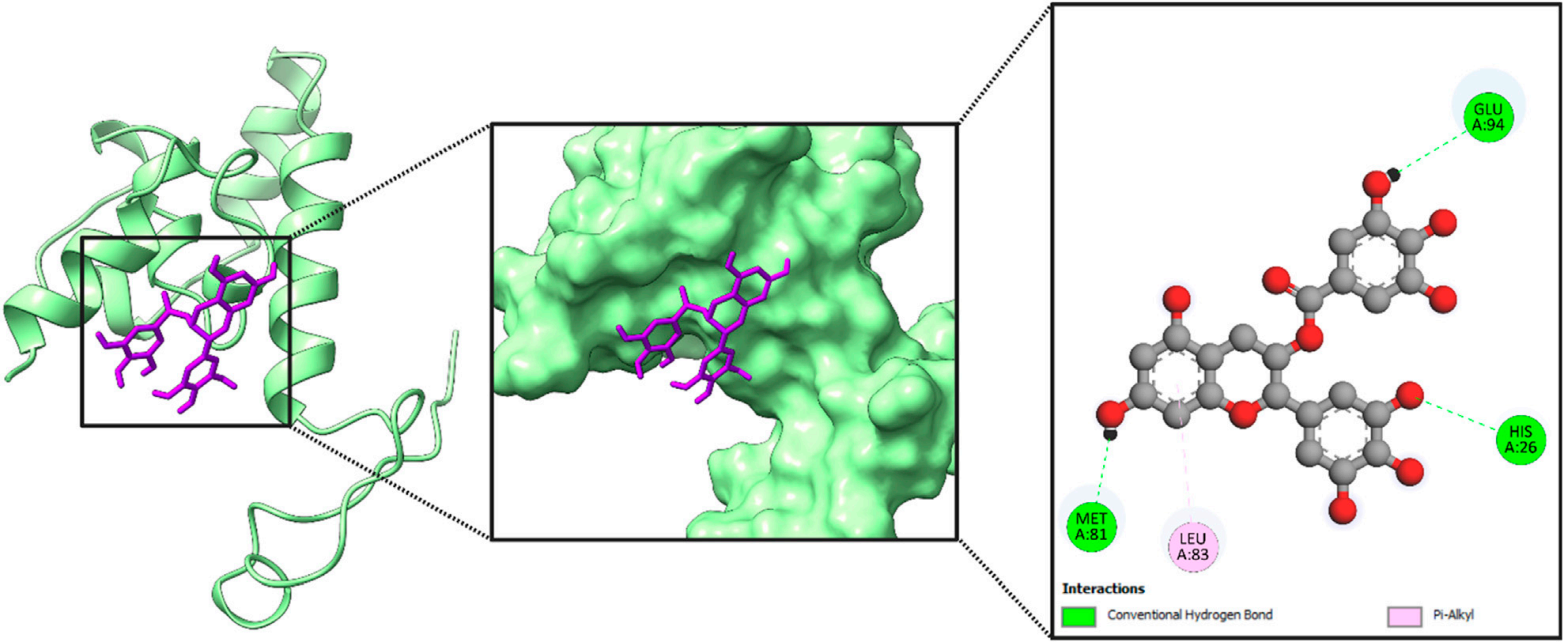
Structural analysis of the compound inside the protein (nucleoprotein) and its interactions with proteins through different amino acids after docking.

**FIGURE 11:**
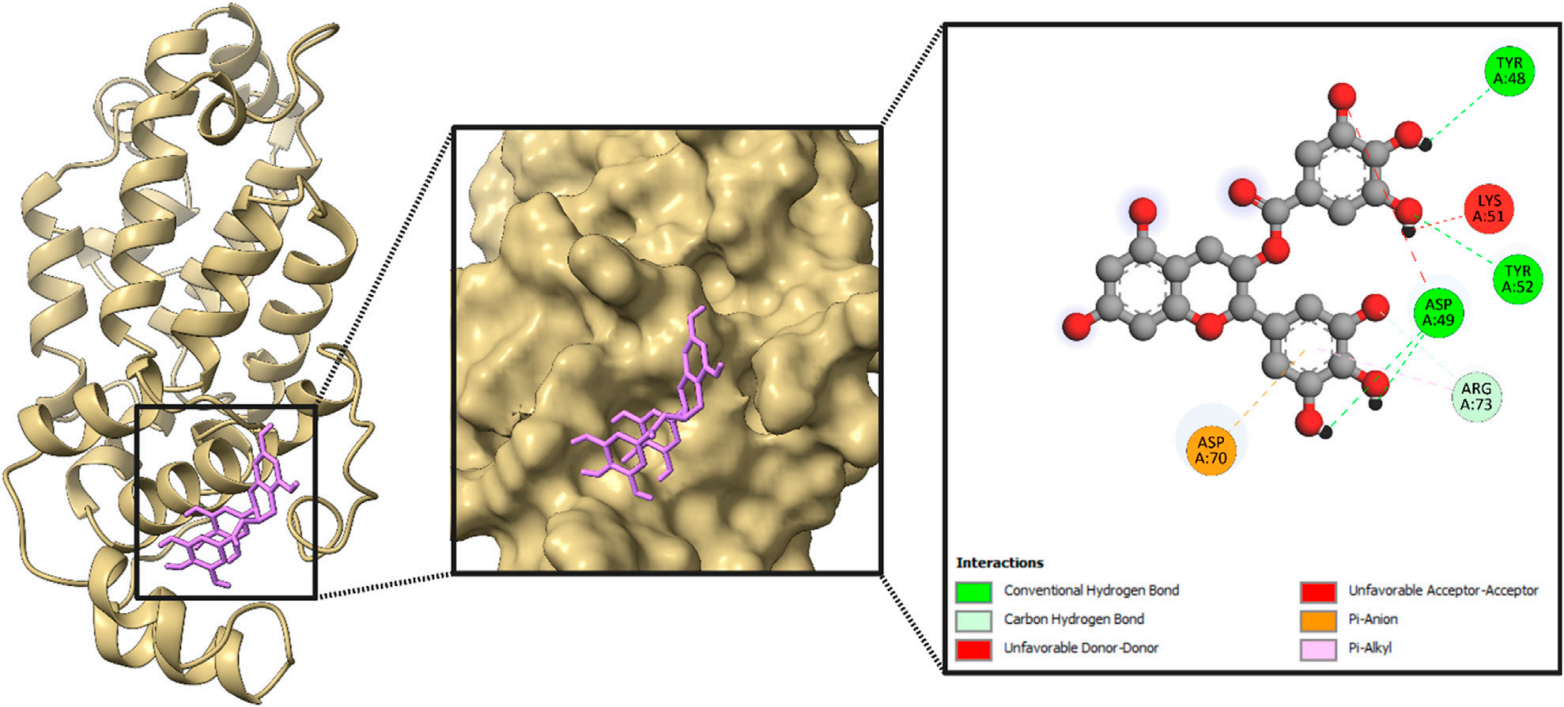
Structural analysis of the compound inside the protein (glycoprotein Gc) and its interactions with proteins through different amino acids after docking.

After an extensive screening process, the protein–ligand interactions of the EGCG compound were analyzed to evaluate the binding affinity of the phytocompound with the viral proteins. The shape and stability of the docked complexes were influenced by hydrophobic interactions and hydrogen bonding. Figures 8–11 present the results, showcasing pose views alongside the 3D and 2D interactions between the proteins and the selected phytocompound.

In Figure 8, the leftmost panel presents the protein’s secondary structure using a ribbon diagram, with the ligand highlighted in purple within a boxed region to indicate its binding position. The middle panel zooms into the ligand binding site, displaying the ligand in purple within the protein’s surface representation, which helps visualize the spatial arrangement within the binding pocket. The rightmost panel provides a detailed view of the ligand’s molecular structure and its interactions with specific amino acids in the protein, including LYS A:23, ILE A:179, PHE A:185, and GLN A:182.

Again, the ligand is highlighted in purple within a boxed region, indicating its binding position in Figure 9. The middle panel offers a closer view of the ligand binding site, illustrating the spatial arrangement of the protein’s surface around the ligand. The rightmost panel provides a detailed molecular schematic of the ligand’s interactions with specific amino acid residues in the protein. The ligand interacts with key amino acids, including ARG A:45, TYR A:102, GLU A:150, LEU A:178, and HIS A:203, contributing to binding stability and specificity. These interactions play a crucial role in determining the docking affinity and potential biological activity of the ligand.

Furthermore, the ligand, highlighted in purple, is positioned within the binding site, indicating its interaction with the protein in Figure 10. The middle panel offers a close-up view of the docking site, illustrating the spatial arrangement of the ligand within the protein’s surface. The rightmost panel presents a detailed schematic of the ligand’s molecular interactions with specific amino acid residues. The ligand engages with key amino acids, including ARG A:67, GLN A:102, HIS A:145, LEU A:189, and TYR A:215, contributing to binding stability and specificity. These interactions provide structural insights into ligand binding, supporting its evaluation as a potential therapeutic candidate.

Moreover, the ligand, highlighted in purple in Figure 11, is situated within the binding site, demonstrating its interactions with key amino acids. The middle panel offers a closer view of the docking region, illustrating the structural arrangement of the protein’s surface surrounding the ligand. The rightmost panel presents a comprehensive molecular interaction map, highlighting the specific interactions between the ligand and the protein. GLN A:54, HIS A:98, TYR A:133, LEU A:176, and ARG A:202 are the key amino acids that bind with the compound.

#### 3.3.3 Binding scores

By yielding the binding energies among the compounds (small molecules) and proteins (large molecules), the docking interactions among the identified compounds and proteins were validated. Redocking with SwissDock helps validate ligand binding predictions by refining molecular interactions and ensuring reproducibility across different docking attempts. The lower the binding affinity is, the stronger the interaction between a compound and protein. The binding results of the targets and compounds are summarized in Tables 1, 2, and all the affinities were less than −5 kcal/mol. Usually, binding values less than −7 kcal/mol indicate strong predicted binding, whereas values less than −5 kcal/mol suggest moderate binding (Pantsar and Poso, 2018).

**TABLE 1:** Binding energy of epigallocatechin-3-gallate with the predicted proteins through PyRx.

Compound name	Compound CID	Protein name			
		RNA-dependent RNA polymerase	Envelopment polyprotein	Nucleoprotein	Glycoprotein Gc
		Binding affinity			
Epigallocatechin-3-gallate (EGCG)	65064	−7.1 kcal/mol	−8.7 kcal/mol	−5.1 kcal/mol	−5.3 kcal/mol

**TABLE 2:** Binding energy of epigallocatechin-3-gallate with the predicted proteins through SwissDock.

Compound name	Compound CID	Protein name			
		RNA-directed RNA polymerase	Envelopment polyprotein	Nucleoprotein	Glycoprotein Gc
		Binding affinity			
Epigallocatechin-3-gallate (EGCG)	65064	−6.9 kcal/mol	−7.5 kcal/mol	−6.1 kcal/mol	−6.7 kcal/mol

**FIGURE 12:**
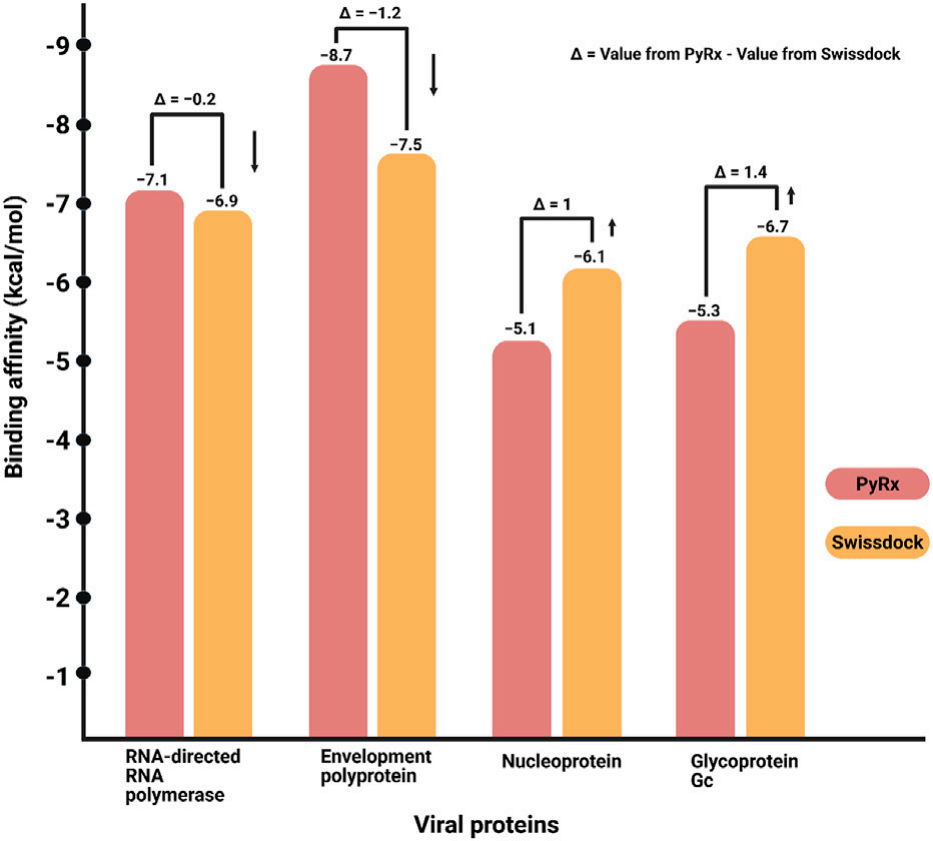
Binding affinity comparison of viral proteins using PyRx and SwissDock. This bar graph illustrates the binding affinities (kcal/mol) of four viral proteins—RNA-dependent RNA polymerase, envelopment polyprotein, nucleoprotein, and glycoprotein Gc—as predicted by two molecular docking platforms: PyRx and SwissDock. Each protein is represented by two bars enclosed within brackets. The left bar corresponds to PyRx docking results, displaying binding affinity predictions, and the right bar represents SwissDock results, highlighting variations in affinity values across docking methodologies. Arrows pointing downward indicate a decrease in affinity values.

The binding affinity analysis of EGCG with four viral proteins—RNA-dependent RNA polymerase, envelopment polyprotein, nucleoprotein, and glycoprotein Gc—was conducted using two different molecular docking platforms: PyRx and SwissDock (Tables 1, 2). The results reveal variations in predicted affinity values across the two tools, highlighting potential methodological differences. For RNA-dependent RNA polymerase, PyRx predicted a binding affinity of −7.1 kcal/mol, while SwissDock estimated −6.9 kcal/mol, showing a minor discrepancy. The envelopment polyprotein displayed a stronger interaction in PyRx (−8.7 kcal/mol) than in SwissDock (−7.5 kcal/mol), suggesting that PyRx may favor more stable ligand binding for this protein. Interestingly, for nucleoprotein, SwissDock reported −6.1 kcal/mol, which indicates a stronger interaction than PyRx’s prediction of −5.1 kcal/mol, revealing an inverse trend. Similarly, glycoprotein Gc showed −6.7 kcal/mol in SwissDock versus −5.3 kcal/mol in PyRx, further emphasizing the differences in scoring algorithms. The accompanying bar graph visualization illustrates these trends, where each protein is represented by two bars, allowing a direct comparison between PyRx and SwissDock predictions. Additionally, downward-pointing arrows highlight cases where binding affinity values decrease (Figure 12).

### 3.4 Identified targets

A total of 214 target genes were retrieved from the OMIM and GeneCards databases after eliminating 110 duplicate entries. These viral target genes were then mapped to the UniProt database under the *Homo sapiens* category, where 7 genes could not be mapped, resulting in 207 successfully mapped targets. These 207 mapped genes were subsequently utilized in further analyses.

### 3.5 Network analysis

**FIGURE 13:**
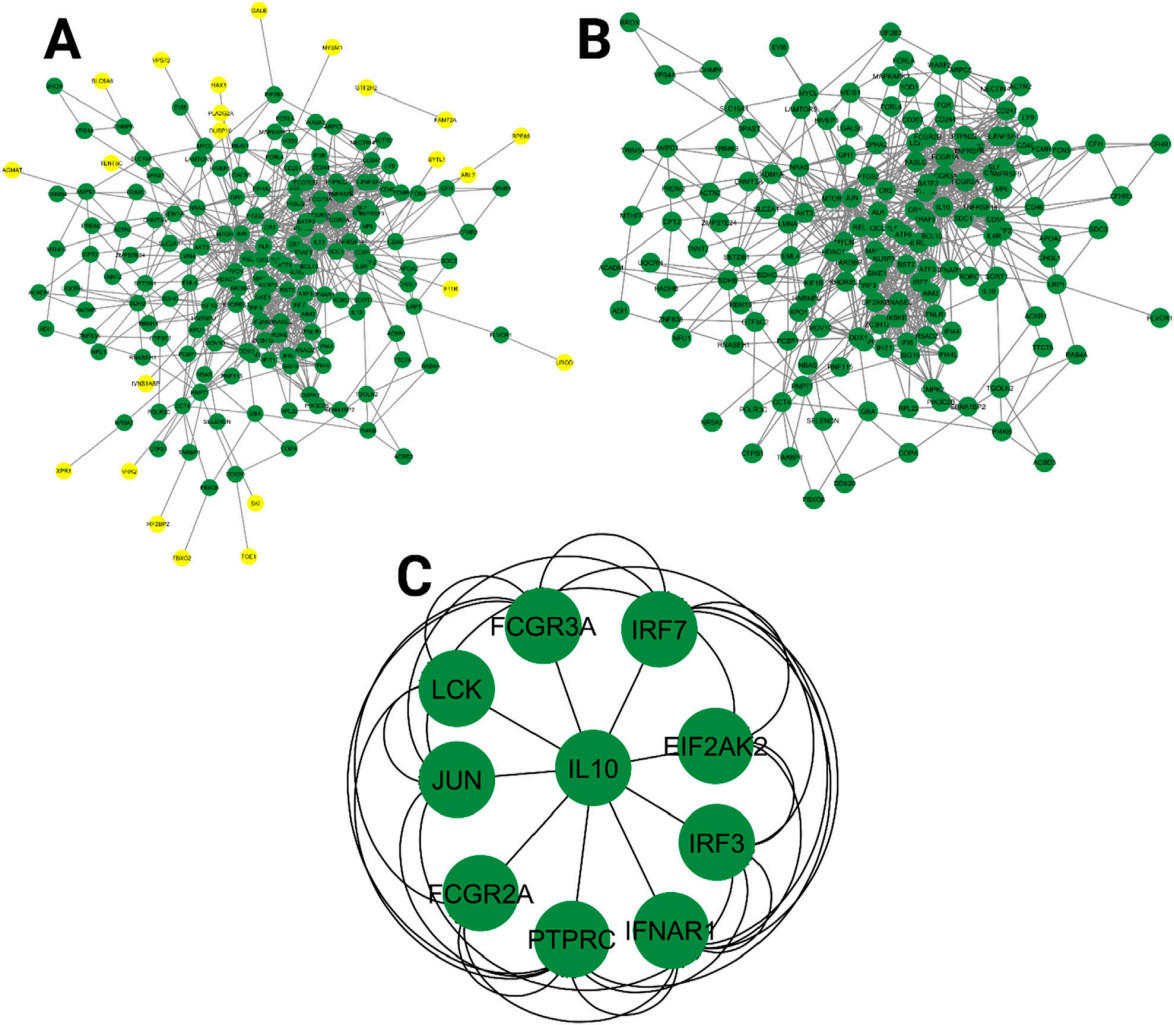
Protein–protein interaction network of Oropouche virus-associated human genes. **(A)** Protein–protein interaction network, with nodes having a value of 1 marked in yellow color. **(B)** Filtered network, where nodes with a degree value of 1 were removed. **(C)** Top 10 targets based on the degree value.

The PPI network constructed for Oropouche virus-associated human genes provides key insights into potential molecular interactions and functional pathways. Figure 13A represents the initial network that has 184 nodes and 808 edges. The nodes signify proteins, and the edges denote interactions, with minimally connected nodes (degree value of 1) highlighted in yellow. Figure 13B presents a filtered version of the network, where nodes with a degree value of 1 have been removed to focus on genes with higher interaction potential that may influence viral pathogenesis or host response mechanisms. This filtered network consists of 161 nodes and 786 edges. Figure 13C highlights the top 10 hub genes, ranked based on the degree value, emphasizing their prominence within the network. We focused on the degree value because a higher degree value indicates more interactions in the network and shows more importance. These hub genes could serve as key regulatory targets or biomarkers involved in the viral response, making them promising candidates for further experimental validation or therapeutic exploration.

### 3.6 GO analysis

The GO and KEGG pathway analysis revealed significant enrichment across biological processes, cellular components, molecular functions, and pathways associated with viral response mechanisms. The analysis yielded a total of 351 entries: 171 related to biological processes (BPs), 39 entries to cellular components (CCs), 56 to molecular functions (MFs), and 85 to KEGG pathways. The top 15 functions for each term were selected based on the enrichment value.

**FIGURE 14:**
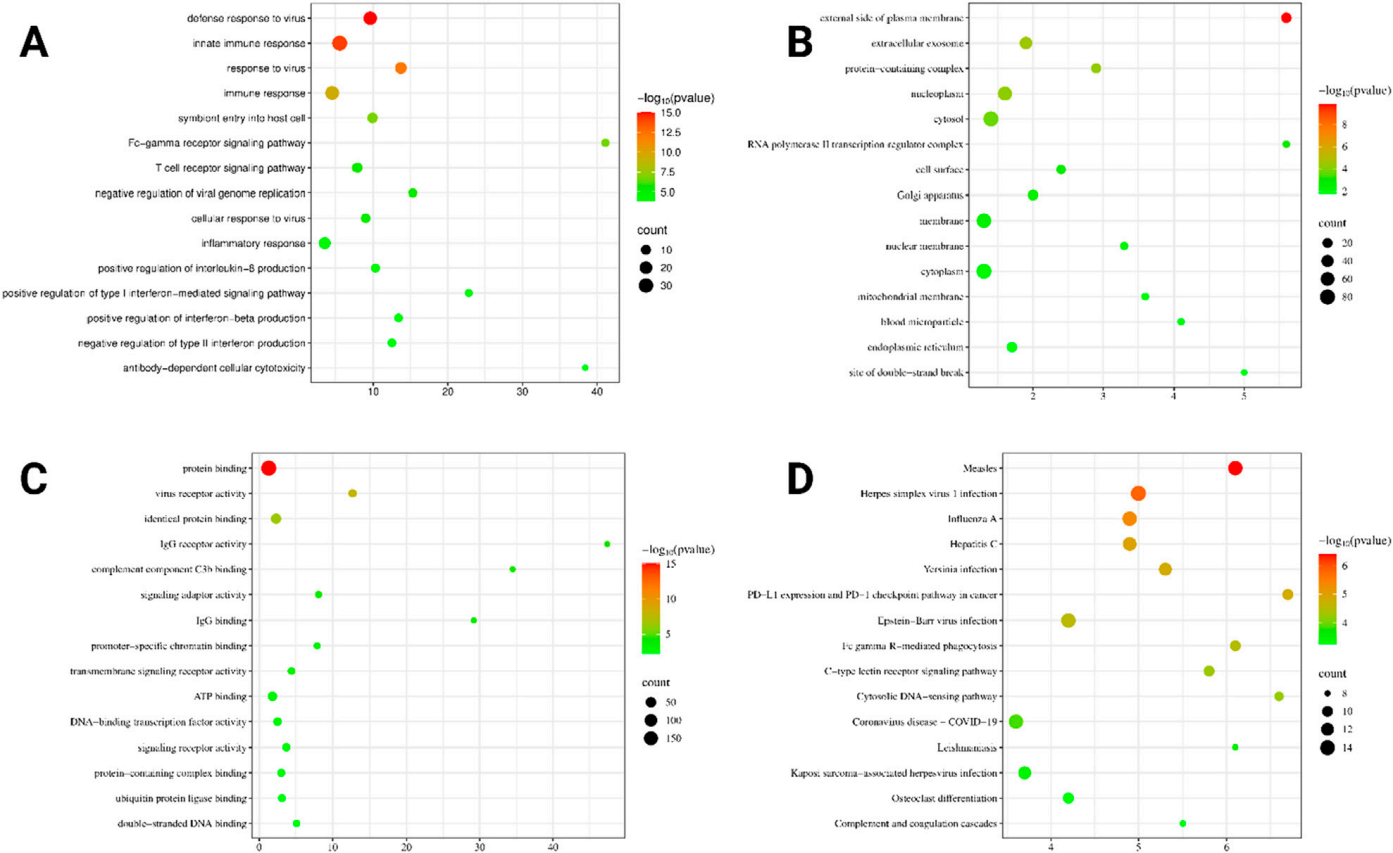
Gene Ontology and KEGG pathway analysis. **(A)** Biological processes in a bubble plot. **(B)** Cellular components in a bubble plot. **(C)** Molecular functions in a bubble plot. **(D)** KEGG pathway in a bubble plot.

In terms of biological processes, enriched terms such as “defense response to virus,” “innate immune response,” and “response to virus” suggest a strong involvement of antiviral defense pathways (Figure 14A). Cellular components, including the external side of the plasma membrane, extracellular exosome, and protein-containing complex, highlight key structural elements that are crucial for cellular communication and immune interactions (Figure 14B). Among molecular functions, protein binding, virus receptor activity, and identical protein binding were prominent, indicating essential interactions for viral recognition and host–pathogen responses (Figure 14C). KEGG pathway enrichment pointed to significant viral infection-related pathways, including measles, herpes simplex virus 1 infection, and influenza A, reinforcing the dataset’s relevance in understanding pathogen-associated molecular processes (Figure 14D). The visualization clearly demonstrates the count distribution and statistical significance of the findings, underscoring the dataset’s strong association with immune response mechanisms.

**FIGURE 15:**
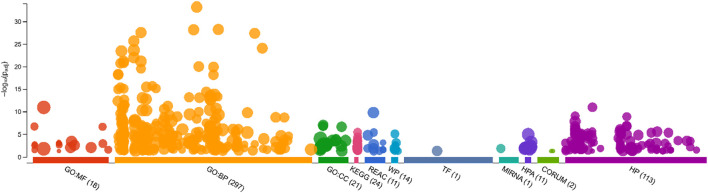
Functional enrichment analysis of genomic data using GeneCloud Omics.

The GeneCloudOmics tool identified a comprehensive set of enriched terms across multiple biological categories, providing insights into Gene Ontology and pathway associations (Figure 15). A total of 297 biological process (GO: BP) terms were found. A total of 24 KEGG pathways and 11 Reactome pathways were enriched, which are critical biological networks. Additionally, the analysis identified one transcription factor (TF) and one microRNA target (MIRNA). The dataset also includes 113 human phenotype (HP) terms, 11 categories from the Human Protein Atlas (HPA), 2 CORUM protein complexes, and 14 WikiPathways (WPs). The statistical significance of these terms is indicated by the -log10(p) scale.

### 3.7 ADMET property

The compound shows a complex ADMET profile with a molecular weight of 458.37 g/mol (Table 3). It has considerable lipophilicity, shown by a minimum log P_o/w_ of 0.95, and variable solubility, classified as soluble by ESOL and SILICOS-IT but only moderately soluble according to Ali. It exhibits low gastrointestinal absorption and poor BBB permeability (Table 3), limiting its ability to cross into the brain. It is neither a P-gp substrate nor an inhibitor of key CYP enzymes, suggesting minimal drug–drug interaction risks. However, the compound violates some drug-likeness requirements, such as Lipinski, Veber, and Muegge, that indicate potential challenges in bioavailability, reflected in its low bioavailability score of 0.17 (Table 3). The compound has been flagged by PAINS and Brenk alerts and has moderate synthetic accessibility (4.20) (Table 3), indicating that while it is a complex molecule, its medicinal chemistry aspects may pose certain challenges.

**TABLE 3:** Properties of EGCG related to drug-likeness evaluation.

Physicochemical property		Water solubility	
Formula	C_22_H_18_O_11_	Log S (ESOL) Solubility Class	−3.56 1.27e-01 mg/mL and 2.76e-04 mol/L Soluble
Molecular weight	458.37 g/mol		
Number of heavy atoms	33		
Number of aromatic heavy atoms	18	Log S (Ali) Solubility Class	−4.91 5.64e-03 mg/mL and 1.23e-05 mol/L Moderately soluble
Fraction Csp3	0.14		
Number of rotatable bonds	4		
Number of H-bond acceptors	11	Log S (SILICOS-IT) Solubility Class	−2.50 1.46e+00 mg/mL and 3.18e-03 mol/L Soluble
Number of H-bond donors	8		
Molar refractivity	112.06		
TPSA	197.37 Å^2^	Pharmacokinetics	
Lipophilicity		GI absorption	Low
Log P_o/w_ (iLOGP)	1.53	BBB permeant	No
Log P_o/w_ (XLOGP3)	1.17	P-gp substrate	No
Log P_o/w_ (WLOGP)	1.91	CYP1A2 inhibitor	No
Log P_o/w_ (MLOGP)	−0.44	CYP2C19 inhibitor	No
Log P_o/w_ (SILICOS-IT)	0.57	CYP2C9 inhibitor	No
Consensus Log P_o/w_	0.95	CYP2D6 inhibitor	No
Medicinal chemistry		CYP3A4 inhibitor	No
PAINS	1 alert catechol_A	Log K_p_ (skin permeation)	−8.27 cm/s
Brenk	1 alert catechol	Drug likeness	
Lead-likeness	No; 1 violation: MW > 350	Lipinski	No; 2 violations: N or O>10 and NH or OH>5
Synthetic accessibility	4.20	Ghose	Yes
		Veber	No; 1 violation: TPSA>140
		Egan	No; 1 violation: TPSA>131.6
		Muegge	No; 3 violations: TPSA>150, H-acc>10, and H-don>5
		Bioavailability	0.17

## 4 Discussion

This study investigated the antiviral potential of EGCG, a green tea-derived catechin, against OROV using computational approaches. Given the absence of specific antiviral therapies or vaccines for OROV and its potential to cause severe neurological conditions, there is a critical need to explore new therapeutic candidates. Our multi-layered analysis integrated protein structure prediction, molecular docking, host–pathogen interaction networks, and ADMET profiling to evaluate EGCG’s potential as a repurposed antiviral agent.

EGCG has been widely studied for its antiviral properties against DNA and RNA viruses (e.g., dengue virus, chikungunya virus, Zika virus, hepatitis C virus, influenza virus, HIV, and Ebola virus) (Xu et al., 2017; Hucke and Bugert, 2020; Loaiza-Cano et al., 2020). Its mechanisms of action generally involve interference with viral entry, replication, and assembly, which share similar pathways for host infection and replication (Li et al., 2020; Zhang X. et al., 2024). This mechanism makes it a useful candidate for suppressing OROV.

A major challenge in OROV research is the lack of experimentally resolved protein structures, which restricts structure-based antiviral design. To overcome this, we utilized AlphaFold2 to predict the 3D structures of four essential viral proteins: RNA-dependent RNA polymerase, envelope polyprotein, nucleoprotein, and glycoprotein Gc. The quality of these models was supported by high plDDT scores (most above 88) and favorable predicted TM-scores (pTM > 0.7 in several cases), indicating high local and global accuracy. Ramachandran plot analysis further confirmed the structural integrity of the predicted models, with over 90% of residues occupying favored regions for most proteins. These predicted structures are crucial as they enabled the identification of active sites and facilitated molecular docking studies. For instance, RNA polymerase, a key enzyme for viral genome replication, showed well-folded catalytic domains consistent with those in other negative-sense RNA viruses (Te Velthuis, 2014). The glycoprotein Gc, essential for host cell entry, also exhibited conserved structural motifs (Gao et al., 2025). By modeling these proteins with confidence, we established a robust structural framework for drug-binding analysis and antiviral screening.

Molecular docking revealed strong binding affinities between EGCG and the predicted viral proteins, particularly the envelope polyprotein and RNA polymerase, with binding energies of −8.7 kcal/mol and −7.1 kcal/mol, respectively, in PyRx simulations. These interactions were supported by hydrogen bonds and hydrophobic contacts at functionally critical residues, suggesting that EGCG may interfere with viral replication and structural assembly. Moderate affinities were observed for glycoprotein Gc and nucleoprotein, implying potential interference with viral entry and nucleocapsid formation.

To explore the host response to OROV infection, we constructed a PPI network using human genes associated with the virus. Out of 214 identified targets, 207 were successfully mapped and visualized in STRING and Cytoscape. cytoHubba analysis identified the top 10 hub genes based on degree centrality, namely, *FCGR3A*, *IRF7*, *EIF2AK2*, *IRF3*, *IFNAR1*, *PTPRC*, *FCGR2A*, *JUN*, *LCK*, and *IL-10*. These genes are closely associated with immune modulation, interferon signaling, and inflammation (Mahaweni et al., 2018; Qing and Liu, 2023; Gan et al., 2024). For instance, IRF7 and IRF3 are pivotal transcription factors regulating type I interferon responses, which are essential for antiviral defense (Daffis et al., 2009; AL Hamrashdi and Brady, 2022). EIF2AK2 (also known as PKR) is a known antiviral mediator activated by viral RNA (Rothenburg et al., 2009), while IFNAR1 encodes a receptor component that is crucial for type-I interferon signaling (Uzé et al., 2007). FCGR3A and FCGR2A mediate antibody-dependent cellular cytotoxicity, underscoring the importance of humoral responses (Paul et al., 2019). PTPRC (CD45) and LCK are essential for T-cell activation (Johnson et al., 2012; Courtney et al., 2019), while JUN is a transcription factor involved in stress and immune signaling (Zhou et al., 2016). IL-10, a key anti-inflammatory cytokine, plays a complex role in regulating immune homeostasis during viral infections (Iyer and Cheng, 2012). The central roles of these hub genes suggest that OROV infection triggers a coordinated immune response involving both antiviral signaling and immune regulation. Gene Ontology and KEGG enrichment further supported these findings, identifying significant pathways such as interferon signaling, cytokine–cytokine receptor interactions, and macrophage-mediated immune responses.

Although EGCG shows promising target engagement, its pharmacokinetic profile poses challenges. ADMET analysis revealed poor blood–brain barrier permeability and limited gastrointestinal absorption. Moreover, EGCG violates multiple drug-likeness rules (e.g., Lipinski and Veber), indicating potential issues in bioavailability and systemic delivery. However, its natural origin, low toxicity, and favorable interaction with target proteins make it a strong candidate for further development, potentially through structural optimization or advanced delivery systems.

EGCG inhibits dengue virus replication by targeting envelope proteins and RNA polymerase (Yamaguchi et al., 2002; Kim et al., 2013). Similarly, its ability to disrupt chikungunya virus entry and replication highlights its versatility as an antiviral agent (Kim et al., 2013; Kaihatsu et al., 2018; Henss et al., 2021). These parallels reinforce the potential of EGCG as a universal antiviral against RNA viruses, including OROV.

This study has several limitations that must be acknowledged. First, all analyses were conducted using *in silico* methods, which, while valuable for hypothesis generation, do not account for complex biological interactions in living systems. Experimental validation is necessary to confirm the antiviral efficacy of EGCG against OROV. Second, due to the unavailability of experimentally resolved OROV protein structures—except for glycoprotein Gc—homology models were generated using AlphaFold2. Although validated through plDDT scores and Ramachandran plots, these structures may not fully capture the native conformations under physiological conditions. Third, EGCG’s pharmacokinetic profile presents challenges, including low gastrointestinal absorption, poor blood–brain barrier permeability, and multiple violations of drug-likeness rules, which could limit its therapeutic applicability. Additionally, host–pathogen gene interaction analysis relied on publicly available databases, which may not encompass all relevant targets. Future studies should include *in vitro* and *in vivo* assays and explore delivery systems or structural modifications to enhance EGCG’s bioavailability and efficacy.

## 5 Conclusion

In this study, computational approaches were employed to evaluate the antiviral potential of EGCG against OROV. Key viral proteins, including RNA-dependent RNA polymerase, envelope polyprotein, nucleoprotein, and glycoprotein Gc, were predicted using AlphaFold2 due to the lack of experimentally resolved structures. Molecular docking revealed strong binding affinities between EGCG and multiple viral proteins, suggesting its potential to interfere with viral replication and entry processes. Protein–protein interaction network analysis identified 10 critical human genes—such as FCGR3A, IRF7, and IFNAR1—involved in immune and antiviral responses. Although pharmacokinetic limitations were observed for EGCG, its natural origin and broad antiviral activity support its potential as a lead compound for further investigation. Experimental validation is recommended to confirm these findings and explore suitable drug delivery strategies.

## Data Availability

The original contributions presented in the study are publicly available. This data can be found here: https://www.uniprot.org/uniprotkb/F5BBK3/entry. Further inquiries can be directed to the corresponding author.
