# Supplementary material for: Epigallocatechin-3-gallate: a multi-target bioactive molecule derived from green tea against Oropouche virus—a computational approach to host–pathogen network modulation
Source: Front Chem. 2025 Jul 3;13:1590498. doi: 10.3389/fchem.2025.1590498 (PMC12268212; doi:10.3389/fchem.2025.1590498)
Supplement: Supplementary file 1 [file DataSheet1.pdf]

Supplementary Material

Supplementary Table S1: List of pathways according to Figure 10

Numerical Value	Connected pathway
**1.0e-04**	**Regulation of I-kappaB kinase/NF-kappaB signaling**
**5.2e-05**	**Positive regulation of I-kappaB kinase/NF-kappaB signaling**
**1.4e-04**	**I-kappaB kinase/NF-kappaB signaling 1.0e-04 Cellular response to exogenous dsRNA**
**4.2e-05**	**Positive regulation of type I interferon-mediated signaling pathway**
**1.3e-04**	**Cellular response to dsRNA 8.4e-07 Interferon-alpha production**
**4.9e-07**	**Positive regulation of interferon-alpha production**
**8.4e-07**	**Regulation of interferon-alpha production**
**1.5e-06**	**Positive regulation of interferon-beta production**
**3.0e-06**	**Interferon-beta production**
**3.0e-06**	**Regulation of interferon-beta production**
**1.0e-05**	**Positive regulation of type I interferon production**

**3.2e-05**	**Regulation of type I interferon production**
**3.2e-05**	**Type I interferon production**
**1.4e-07**	**Type I interferon signaling pathway**
**1.4e-07**	**Response to type I interferon**
**1.4e-07**	**Cellular response to type I interferon**
**1.4e-06**	**Defense response to virus**
**1.4e-06**	**Defense response to symbiont**
**3.0e-06**	**Response to virus**
**5.2e-05**	**Cytokine production**
**5.2e-05**	**Regulation of cytokine production**
**5.6e-05**	**Cytokine-mediated signaling pathway**
**1.0e-04**	**Viral process**
**1.3e-04**	**Innate immune response**
**3.7e-05**	**MDA-5 signaling pathway**
**3.3e-05**	**Macrophage apoptotic process**
**4.4e-05**	**Regulation of dendritic cell cytokine production**
**4.4e-05**	**Dendritic cell cytokine production**
